# Evaluation of the predictive role of neutrophil/lymphocyte ratio in the diagnosis of lymphoma in patients with asymptomatic and ısolated cervical lymphadenopathy

**DOI:** 10.1016/j.bjorl.2020.06.012

**Published:** 2020-08-01

**Authors:** Mehmet Erkan Kaplama, Ahmet Kürşad Güneş, Burak Erden

**Affiliations:** a Şanlıurfa Mehmet Akif İnan Training and Research Hospital, Department of Otorhinolaryngology, Şanlıurfa, Turkey; b Ankara City Hospital, Department of Hematology, Ankara, Turkey; c Mersin City Training and Research Hospital, Department of Otorhinolaryngology, Mersin, Turkey

**Keywords:** Lymphoma, non-hodgkin, Hodgkin disease, Predictive value of tests, Neutrophil lymphocyte ratio

## Abstract

**Introduction:**

The diagnostic approach to patients with isolated asymptomatic cervical lymphadenopathy varies between excisional biopsy and follow-up. When the anamnesis, physical examination, laboratory and imaging findings are not sufficient to identify the etiology, an excisional biopsy is performed for the differential diagnosis between early-stage lymphoma and infectious or reactive causes. If the excisional biopsy, which may have some complications, is not performed, it may delay the diagnosis of lymphoma. This diagnostic challenge could be avoided by predictive markers.

**Objectives:**

This study was planned to determine the predictive value of neutrophil/lymphocyte ratio in the diagnosis of Hodgkin and non-Hodgkin limphoma in patients with asymptomatic, isolated cervical limphadenopathy and underwent excisional biopsy.

**Methods:**

A total of 90 patients between the years 2016 − 2019 admitted to our clinics due to asymptomatic isolated cervical lymphadenopathy, present in at least 4 weeks with lympho nodes in pathological dimensions persisting in the cervical region, were included to our study. An excisional lympho node biopsy was performed in all 90 patients.

**Results:**

Of the 90 patients who underwent excisional biopsy; 34 were diagnosed as reactive lymphadenopathy 30 were non-Hodgkin linphoma, and 26 were Hodgkin linphoma. A total of 56 (62.2%) patients were diagnosed as lymphoma, either Hodgkin or non-Hodgkin, while 34 patients (38.8%) were diagnosed as reactive lymphadenopathy. The median age, total whiteblood count, neutrophil count of the lymphoma groups were significantly higher than reactive lymphadenopathy group, whereas the lymphocyte count was significantly lower in the lymphoma patients. The median neutrophil/ lymphocyte ratio was 1.7 in the reactive lymphadenopathy group, 3.5 in the non-Hodgkin limphoma group, and 3.0 in the Hodgkin limphoma group (*p* <  0.001).

**Conclusion:**

According to the results of our study, neutrophil/lymphocyte ratio was significantly higher in patients who were admitted with isolated asymptomatic lymphadenopathy and were diagnosed with lymphoma, and who were diagnosed with early-stage Hodgkin and non- Hodgkin lymphoma compared to those who were found to have reactive lymphadenopathy. Neutrophil/lymphocyte ratio, which is a low-cost, fast and easy-to-access test, has a predictive value in the diagnosis of lymphoma in patients with asymptomatic lymphadenopathy.

## Introduction

Lymphomas are malignant tumors arising from lymphoid tissue. According to the WHO 2016 classification, they are divided into two main groups as Hodgkin and non-Hodgkin lymphomas. Lymphadenopathy (LAP) in the cervical region is painless in 60–80% of Hodgkin lymphoma cases.^1^ While 20% of B symptoms are seen in early-stage of HL, this rate may increase in advanced-stage disease. In other words, approximately 80% of early-stage Hodgkin patients with supradiaphragmatic involvement may be asymptomatic.^2^ Non-Hodgkin's lymphoma’s (NHL) clinical manifestations vary according to the histological subtype. B symptoms may be observed in 40% of NHL cases with aggressive histological subtype, while indolent progressive forms the symptoms are seen in 20%−25%.^3^ Diagnosis of lymphoma is made by histopathological examination. While fine needle aspiration biopsy (FNAB) can differentiate carcinoma and tumors that cause lympho node metastasis, it is not sufficient to confirm the diagnosis of lymphoma, and excisional biopsy is required for histological subtyping. Although FNAB or blind biopsies are beneficial in relapsed disease, false-negative results of FNAB in newly- diagnosed patients create a diagnostic challenge.

Neutrophil lymphocyte ratio (NLR) is a parameter that indicates a systemic inflammatory status. There have been many developments regarding the correlation between cancer and inflammation.^4^ Publications are indicating that NLR can be a prognostic marker of mortality in important cardiac events, in several solid tumors, and in hematological malignancies.^5^, ^6^, ^7^, ^8^, ^9^, ^10^

Regarding isolated asymptomatic cervical LAP, in cases where anamnesis, physical examination, imaging findings and the etiological cause can not be determined, an excisional lympho node biopsy can differentiate early-stage lymphomas from infectious or reactive causes. The surgical procedure may be risky due to both, the surgeon’s experience and the complex anatomy of the region, but for these reasons, the diagnosis of lymphoma can be missed in patients who are not operated. Therefore, in patients with asymptomatic LAP, some predictive tests are needed to support the diagnosis of lymphoma before the biopsy.

This retrospective study was planned to determine the predictive value of NLR in the diagnosis of HL and NHL in patients who presented with asymptomatic isolated cervical LAP and underwent excisional biopsy.

## Methods

### Ethics committee approval

Thestudy was conducted by the principles of the Helsinki declaration and was approved by the Harran University corporate ethics committee with permission from HRU/20.02.08 dated 27/01/2020.

### Patient characteristics

A total of 90 patients between the years 2016–2019 who were admitted to Sanliurfa Mehmet Akif Inan Training and Research Hospital otorhinolaryngology and hematology clinics due to asymptomatic isolated cervical LAP, presenting with persisting and enlarged lympho nodes for least 4 weeks in the cervical region, were included in our retrospective study. Patients who were at least 18 years old, with no etiological cause by history, but in whom physical examination and laboratory findings suggested the suspicion of lymphoma were included. Excisional lympho node biopsy were performed in all 90 patients. The patients underwent a complete ear, nose and throat examination, including ear examination, endoscopic examination of the nasal cavities and nasopharynx, oropharynx, hypopharynx, larynx palpation, endoscopic examinations, scalp facial skin examination, thyroid examination and neck palpation. Included patients were submitted to a full examination with a high-definition, HDR (High Dynamic Range) and special imaging modes (SIM) features that the endoscopic systems (Richard Wolf, ENDOCAM Logic HD Knittlingen, Germany). Endoscopic examination of the nasopharynx and indirectly examination through laryngoscopic investigation determined that there were no mucosal lesions. Erytrocyte sedimentation rate and C-reactive protein (CRP) laboratory exams were also requested. All patients were tested for toxoplasma, cytomegalovirus (CMV), rubella, herpes and Ebstein -Barr (EBV) IgM and IgG antibodies. In all patients, serologic IgM antibody results were negative and the presence of acute viral infection was excluded. Also, anti-HIV, anti-HCV and HbsAg tests were negative in all patients. Specific serological tests such as brucella, tularemia, syphilis, CMV-PCR and EBV PCR and parvovirus B19 were only requested for potentially suspicious patients and possible viral/bacterial infectious diseases were excluded. In addition, if autoimmune disease was suspected, antinuclear antibody (ANA) and rheumatoid factor (RF) levels were checked to exclude rheumatologic disorders. A tuberculin skin test (PPD) was requested in patients with a history of tuberculosis; and patients diagnosed with tuberculosis were excluded from the study. All patients were given antibiotic therapy for 14 days for possible infectious agents. Patients with a solid mass were also excluded from the study. Considering that unilateral neck masses and supraclavicular mass may be metastatic, chest diseases, internal medicine, and oncology consultations were requested, and patients without suspicion of lymphoma were excluded. Patients who had absolute lymphocytosis (> 5 × 10^3^μL) in the complete blood count and whose flow cytometric analysis of CLL showed follicular lymphoma, or the leukemic form of marginal zone lymphoma were not included in the study; patients who received corticosteroid therapy in the past 6 months for any reason were not included in the study. Blood samples of the patients were taken from the peripheral veins at the time of admission. Hemograms were measured using the Abbott Architect c-8000 system. Leukocyte, neutrophil and lymphocyte counts of all patients were confirmed by peripheral smear. NLR was calculated by dividing the absolute neutrophil count in the whole blood count by the absolute lymphocyte count, and the ratios were confirmed by peripheral smear. Pathology preparations of all patients were evaluated by the same pathologist and the patients were included into three groups as reactive lymphadenopathy (RAL), HL and NHL.

### Surgical technique

Biopsies of the patients were performed either under local anesthesia, sedation anesthesia or general anesthesia according to the patient preference and the decision of the surgeon. Before the operation, the surgical field was cleaned, and shaved if necessary. The patients were placed in a supine position with their heads facing the opposite side. The head was placed in extension with a support under the shoulders and neck. The surgical site was sterilized with iodine solution and covered with sterile drapes. Adrenaline (0.025 mg/mL) and lidocaine (20 mg/mL) were injected with a 22 gauge syringe to reduce bleeding during the incision. Then, a small incision was made over the lympho node to be removed with #15 scalpel and the specimen removed in one piece. Once the skin and subcutaneous adipose tissue were overcome, if vascular structures and nerves in the surgical field could be preserved, they were protected with a farabeuf retractor. If the vascular structures could not be preserved, they were displaced from the surgical field and sutured with a Vicril binding suture (Pegelak®, Doğsan Ankara, Turkey) or cauterized with bipolar cautery (Petaş Petkot 500S, Ankara, Turkey); and finally, the lympho node capsule was reached. Nerves were always displaced and protected.

With the help of a kelly forceps the lympho node capsule, was totally removed with blunt dissection. After the bleeding control with bipolar cautery, 4.0 vicril rapidle (Pegelak® Rapid, Doğsan Ankara, Turkey) and 4.0 − 5.0 prolene (Propylene®, Doğsan Ankara, Turkey) were used subcutaneouly. Drains were not applied. Finally, pressure dressings with thiocilline pomade were applyed to the area. All patients were discharged after a 6 − 8 h observation. If the patients were not observed after the pressure, dressings were opened the next day, the surgical site was packed with a small gauze and no more dressings were applied from the 2nd postoperative day. Patients were discharge with oral amoxicillin-clavulanate for one week, and paracetamol for pain when needed. If no problem was observed after 1 week, the skin sutures were removed.

### Statistical analysis

Statistical analyzers were performed with SPSS version 20 (IBM Corp. in Armonk, NY). Kolmogorov-Smirnov and Shapiro-Wilk tests were used to evaluate the distribution of the data. Descriptive data are presented as frequencies (n) and percentages (%) for categorical variables, and median with interquartile range (IQR) for non-normally distributed numerical variables. Pearson Chi-Square test was used for comparing categorical variables, Kruskal Wallis test was used for comparing numerical variables among groups. Dunn’s Post-hoc Test was used for post-hoc pairwise comparison of the groups; *p* <  0.05 was considered as statistically significant.

## Results

This comparative study was conducted with 90 patients who were admitted to our otorhinolaryngology and hematology clinic with cervical LAP, and who underwent diagnostic excisional lympho node biopsy, and were diagnosed with RAL, NHL or HL. Of the patients, 34 were RAL, 30 were NHL, and 26 were HL. A total of 56 patients were diagnosed with lymphoma (62.2%) either HL or NHL, while 34 patients (38.8%) were diagnosed with RAL. The median age was 27.0 years in the RAL group, 49.0 years in the NHL group, and 31.0 years in the HL group. There was a statistically significant difference among the groups (*p* =  0.016) (Table 1). In the pairwise comparisons of age, there was a statistically significant difference only between RAL and NHL groups (*p* =  0.016).

**Table 1:** Demographics of the patients.

Variable	RAL (n = 34)	NHL (n = 30)	HL (n = 26)	*p*
Age (year), median (IQR)	27.0 (20.5 − 41.0)	49.0 (41.0 − 58.0)	31.0 (24.5 − 44.5)	0.016^a^
Sex, n (%)				0.064^b^
Female	20 (58.8)	6 (20.0)	8 (30.8)	
Male	14 (41.2)	24 (80.0)	18 (69.2)	

RAL, Reactive lymphadenopathy; NHL, Non-Hodgkin lymphoma; HL, Hodgkin lymphoma; IQR, Interquartile range.

a Kruskal-Wallis test was used.

b Pearson Chi-square test was used.

The median leukocyte count was 5.7 × 10^3^μL in the RAL group, 12.3 × 10^3^ μL in the NHL group, and 6.7 × 10^3^μL in the HL group. This difference was statistically significant (*p* =  0.016). The median neutrophil count was 3.8×10^3^μL in the RAL group, 9.2 × 10^3^μL in the NHL group, and 5.3 × 10^3^ μL in the HL group. The difference between the groups was statistically significant (*p* =  0.001). The median lymphocyte count was 2.1×10^3^μL in the RAL group, 2.8 × 10^3^μL in the NHL group, and 1.7 × 10^3^μL in the HL group. There was a statistically significant difference for lymphocyte count between groups (*p* =  0.033). The median platelet count was 293.0×10^3^μL in the RAL group, 275.0 × 10^3^μL in the NHL group, and 319.0 × 10^3^μL in the HL group. This difference was not statistically significant (*p* =  0.715). The median NLR was 1.7 in the RAL group, 3.5 in the NHL group, and 3.0 in the HL group. This difference was statistically significant (*p* <  0.001). The median PLR was 109.3 in the RAL group, 128.9 in the NHL group, and 173.9 in the HL group. The difference in PLR was not statistically significant between groups (*p* =  0.051) (Table 2).

**Table 2:** Comparison of the complete blood count variables between the study groups.

Variable	Median (IQR)			*p*^a^
	RAL (n = 34)	NHL (n = 30)	HL (n = 26)	
Leukocyte (×10^3^μL)	5.7 (4.8 − 8.3)	12.3 (8.7 − 17.9)	6.7 (4.9 − 8.3)	0.016
Neutrophil (×10^3^μL)	3.8 (2.7 − 4.4)	9.2 (4.3 − 12.9)	5.3 (4.4 − 5.9)	0.001
Lymphocyte (×10^3^μL)	2.1 (1.6 − 3.2)	2.8 (1.8 − 3.8)	1.7 (1.3 − 2.3)	0.033
Platelet (×10^3^μL)	293.0 (256.0 − 306.5)	275.0 (188.0 − 391.0)	319.0 (243.0 − 356.5)	0.715
MPV (fL)	8.4 (6.9 − 9.2)	7.3 (6.3 − 8.5)	7.1 (6.9 − 7.9)	0.135
NLR	1.7 (1.3 − 1.9)	3.5 (2.3 − 4.8)	3.0 (2.4 − 3.9)	<0.001
PLR	109.3 (91.7 − 166.2)	128.9 (40.4 − 165.5)	173.9 (139.2 − 249.2)	0.051

IQR, Interquartile range; RAL, Reactive lymphadenopathy; NHL, Non-Hodgkin lymphoma; HL, Hodgkin lymphoma; MPV, Mean llatelet volume; NLR, Neutrophil lymphocyte ratio; PLR, Platelet to lymphocyte ratio.

a Kruskal-Wallis test was used.

**Figure 1:**
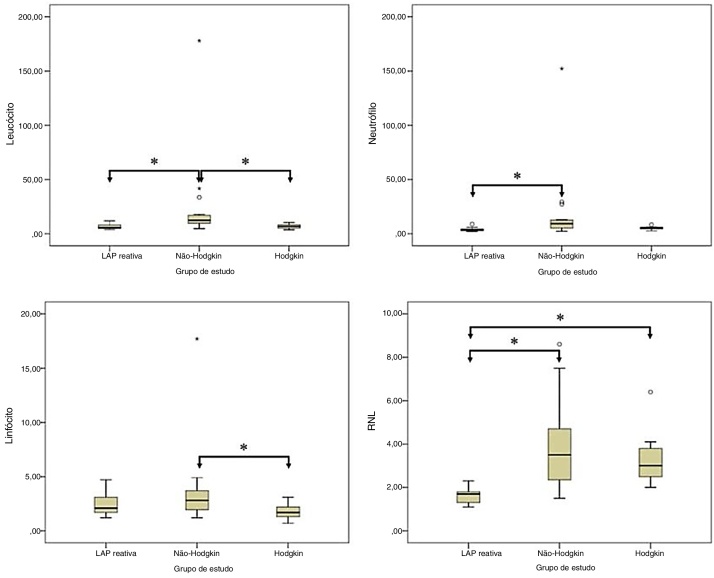
Comparison of (a) leukocyte, (b) neutrophil, (c) lymphocyte, and (d) NLR distributions among the study groups. Note: *p < 0.05.

In the pairwise comparison, there were statistically significant differences in the lymphocyte count between RAL and NHL groups and between NHL and HL groups (*p* =  0.002, and p = 0.004, respectively, in the neutrophil count between RAL and NHL groups (*p* <  0.001); and in the lymphocyte count between NHL and HL groups (*p* =  0.030). Moreover, the NLR was statistically signiﬁcantly higher in NHL and in HL groups than in RAL group (*p* <  0.001 and *p* <  0.001, respectively) (Fig. 1).

## Discussion

Head and neck masses are seen quite frequently in all age groups. There are complexities and controversies about the approaches to the differential diagnosis; therefore a systematic approach is required. There are many diseases in the differential diagnosis of head and neck masses, which includes a wide spectrum of illnesses from infection-related benign masses to congenital masses, from traumatic causes to malignant masses. Although medical history, physical examination, laboratory and radiological examinations guide the diagnosis of these masses, it is often possible and preferable to make a definitive diagnosis with the histopathological examination of the tissue samples obtained surgically.^11^ Fine needle aspiration biopsy is an inexpensive, reliable and simple method that can be used for diagnosis in neck masses. The most known disadvantages of this examination are that it requires experienced cytologists, it does not give results in the masses from the region where the primary disease is present.^12^ It is a known fact that FNAB is a highly accurate diagnostic method for head and neck tumors, except in lymphomas. This specificity, sensitivity and accuracy of diagnosis in other head and neck masses lose their validity when it comes to lymphoma.^13^ Considering false negativity, a negative FNAB does not rule out the lymphoma.^14^ The benefits of FNAB for diagnosis are generally limited to subtyping. In these cases, excisional biopsy is needed because it will change the form of treatment.^15^

LAP is the term used to define the circumstances in which lympho nodes become abnormal in size, consistency, and number. In LAP etiology, infectious causes, autoimmune diseases, solid organ tumors, hematological malignancies, drugs and systemic diseases should be included.^16^ LAP can be localized or generalized. Since the cervical region is lymphoid-rich, both RAL due to infectious causes and hematological malignancies such as leukemia and lymphoma can often originate from this region. In fact, in some early-stage lymphomas, the involvement area can only be the cervical region.

Etiological approaches to patients with LAP, include a thorough history and physical examination, since it is essential to obtain some clues about the underlying disease. Time of the existence of the lympho node, growth rate, associate infection findings, presence of underlying diseases, drugs used, previous radiation exposure (prior radiotherapy or radiation exposure), itching, pain in the lympho node, excess alcohol intake and the presence of other symptoms (eg. fever, night sweats and at least 10% loss of body weight in the last 6 months) may give some etiological clues.^17^, ^18^, ^19^ Also, on physical examination, the lympho node diameter, structure, whether it is fixed or not, tenderness/fixation, presence of accompanying infection findings, presence of hepato-splenomegaly can give an clue about the lympho node etiology. However, some of the patients may be asymptomatic and present with isolated cervical LAP. The presence of LAP larger than 1 cm in the cervical region and present for at least 4 weeks requires further investigation.^20^, ^21^

Studies have shown that large cervical lympho nodes for 4 or more weeks can have a role in the development of cancer/inflammation and in the survival of cancer patients.^4^ There is also evidence that neutrophils, which are the components of cancer-related inflammation, have a role in tumor progression and metastasis development.^22^ In addition, it has been shown that lymphocytes in the tumor tissue microenvironment are tumor suppressors and in contrast to neutrophils, suppress inflammation and tumorogenesis.^23^ Following these advances in malignant transformation, studies on NLR, which can be an indicator for both an increased neutrophil count and suppressed lymphocytes, are thought to show the balance between pro-inflammatory status and anti-tumor immunity, have accelerated. The prognostic value of NLR, which is one of the indicators of systemic inflammation, in many cancer types and hematological malignancies, especially lymphoma subtypes, has been validated.^6^, ^7^, ^8^, ^9^, ^10^

There are studies on predicting malignant lymphoma with biochemical markers before performing a LAP biopsy. Matsumoto et al., Serum IL-2R levels, another indicator of the inflammatory process, have been shown to be higher in patients with lymphoma than the RAL group.^24^ In another study, Tjusi et al. noted that before LAP biopsy, serum thymidine kinase and IL-2R levels were found to be higher in the malignant lymphoma group.^25^ However, one study in the literature investigated the diagnostic predictive value of NLR before excisional biopsy in HL patients.^20^ In our study, we compared NLR according to the RAL group in patients diagnosed with NHL and HL as a result of the biopsy.

In our study, patients who were treated for asymptomatic isolated cervical LAP and who underwent excisional LAP biopsy were included and examined in three groups according to the pathology result. The mean age of patients diagnosed with HL and NHL was higher than the RAL group. In the literature, studies with large patient populations have shown that similar to our study for LAP etiology, increased malignancy rates occur with increasing age.^21^, ^22^, ^23^, ^24^, ^25^

In our study, male predominance was detected according to the RAL group, although it was not statistically significant in patients with HL and NHL. The studies in the literature, both HL and NHL has been shown to be more common in men, in line with our study.^26^

Both total leukocyte count (WBC) and neutrophil count in NHL and HL groups were significantly higher than in the RAL group. The median lymphocyte count was also significantly higher in the NHL group than in the RAL and HL groups. The median NLR was 1.7 in the RAL group, 1.7 (1.3 − 1.69), 3 in the HL group (2.4 − 3.9), and 3,5 in the NHL group (2.3 − 4.8). Before the LAP excision, NLR was found to be significantly higher in the lymphoma group than in the RAL group (*p* <  0.001).

In a study about normal values ​​of NLR investigating what cut-off value should be taken, the average NLR value was found to be 1.65 in 413 healthy adults.^27^ NLR cut-off values ​​were determined differently in different lymphoma subtypes. In angioimmunoblastic T cell lymphoma in 2020, NLR ≥ 2.2 has been shown to be related with a poor prognosis.^28^ In NK-T cell NHL, NLR ≥ 3,6 was associated with a poor prognosis.^29^ In a meta-analysis of 2297 cases and 9 major studies related to the prognostic role of NLR in diffuse large B cell NHL, the effect of high NLR on total survival (OS) and progression-free survival (PFS) was demonstrated. The NLR cut-off value was determined as < 3 in 7 of these studies.^30^ In our study, similarly to the literature, the median NLR HL arm was 3, and the NHL arm was 3.5.

There are seven parameters in the international prognostic score (IPS) system, which is the most frequently used in determining the prognosis of advanced-stage HL. Two of these 7 parameters, which have been shown to be associated with poor prognosis, include total WBC > 15,000 micro L,while another is the absolute lymphocyte count < 600 micro L.^31^ One of the best markers of these two parameters in IPS showing increased white blood cell count and decreased lymphocyte count is NLR. It was found that the height of NLR in HL is associated with poor prognosis.^32^

There are also studies on the diagnostic value of NLR in cancer disease. In the study of Seretis et al., related to malignancy and NLR in thyroid nodules, as a result of the biopsy, NLR 1.8 − 1.9 was detected in the group with a benign thyroid nodule, while NLR 3 − 3.1 was detected in patients with thyroid papillary cancer and NLR thyroid nodules It has been shown to predominantly predict its malignancy.^33^ Kılıçkap et al. found that in cases where the endoscopic biopsy was suspected to be colon cancer, NLR > 2.02 before the biopsy was associated with the diagnosis of colon cancer.^34^

There is a study by Çolak et al. about the diagnostic predictive value of NLR in patients with LAP. In 46 patients with asymptomatic cervical LAP, NLR was calculated prior to excisional biopsy. In patients diagnosed with HL, the average NLR was 5.8, while the RAL group was found to be NLR 2.6. Before the excisional biopsy, the NLR was found to be significantly higher in the Hodgkin lymphoma group than in the RAL group.^20^ In our study, the median NLR 3 was detected in the HL arm. In this study, the detection of NLR in the HL group higher than our study was thought to be due to the fact thatt some of the cases in this study may be at the advanced stage. All of the patients in our study are cases of early stage lymphoma diagnosed by isolated cervical LAP. Another study showed that NLR is found to be lower in the early-stage HL than in the advanced stage.^35^

The relationship between NLR and prognosis in many solid tumors and lymphoma subtypes, and its predictive role in the diagnosis of some cancer types, were investigated. However, there is no study on the diagnostic value of NLR in NHL patients in patients undergoing excisional LAP biopsy with a pre-diagnosis of lymphoma. Although there is a study related to the role of NLR in the diagnosis of HL, the role of NLR in early-stage HL, which came with isolated cervical LAP, as in our case series, has not been studied. In our study, there are some limitations such as retrospective design, the low number of cases and inability to analyze specifically for non-Hodgkin lymphoma subtypes. The predictive value of NLR in the diagnosis of lymphoma should be supported by prospective studies with a greater number of cases in a multicenter study..

## Conclusion

According to the results of our study, NLR was significantly higher in patients who were admitted with isolated asymptomatic LAP and who were diagnosed with lymphoma, and who were diagnosed with early-stage HL and NHL compared to those who were found to have RAL. NLR, which is a low-cost, fast and easy-to-access test, has a predictive value in the diagnosis of lymphoma in patients with asymptomatic LAP.

## Conflicts of interest

The authors declare no conflicts of interest.
